# Musculoskeletal biomechanics of patients with or without adjacent segment degeneration after spinal fusion

**DOI:** 10.1186/s12891-021-04916-z

**Published:** 2021-12-13

**Authors:** Mazda Farshad, Pascal Raffael Furrer, Florian Wanivenhaus, Lukas Urbanschitz, Marco Senteler

**Affiliations:** 1 Department of Orthopaedics, Balgrist University Hospital, University of Zurich, Forchstrasse 340, 8008 Zurich, Switzerland; 2 Institute for Biomechanics, ETH Zürich, Leopold-Ruzicka-Weg 4, 8093 Zurich, Switzerland

**Keywords:** Adjacent segment disease, Spinal fusion, Biomechanical modeling, Patient-specific biomechanical simulation, Preoperative planning, Sagittal alignment

## Abstract

**Study design:**

A retrospective, single center, case-control study was performed.

**Objective:**

The present study employed patient-specific biomechanical modeling to find potential biomechanical differences after spinal fusion at L4/5 in patients with and without subsequent development of adjacent segment disease (ASD).

**Methods:**

The study population comprised patients who underwent primary spinal fusion at L4/5 and were either asymptomatic during > 4 years of follow-up (CTRL; *n* = 18) or underwent revision surgery for ASD at L3/4 (*n* = 20). Landmarks were annotated on preoperative and follow-up lateral radiographs, and specific musculoskeletal models were created using a custom-built modeling pipeline. Simulated spinal muscle activation and lumbar intervertebral shear loads in unfused segments were analyzed in upright standing and forward flexion. Differences between the pre- and postoperative conditions were computed for each patient.

**Results:**

The average postoperative muscle activity in the upright standing posture was 88.4% of the preoperative activity in the CTRL group (*p* <  0.0001), but did not significantly change from pre- to postoperatively in the ASD group (98.0%). The average shear load magnitude at the epifusional joint L3/4 during upright standing increased from pre- to postoperatively in the ASD group (+ 3.9 N, +/− 17.4 (*n* = 18)), but decreased in the CTRL group (− 4.6 N, +/− 23.3 (*n* = 20); *p* <  0.001).

**Conclusion:**

Patient-specific biomechanical simulation revealed that spinal fusion surgery resulted in greater shear load magnitude and muscle activation and therefore greater forces at the epifusional segment in those with ASD compared with those without ASD. This is a first report of patient-specific disc load and muscle force calculation with predictive merits for ASD.

## Introduction

Adjacent segment disease (ASD) is a major complication of spinal fusion surgery, with a reported incidence of 37.4% within 5 to 20 years postoperatively [1]. Numerous studies have identified potential risk factors for ASD, such as age, sex, osteoporosis, number of fused segments, and laminectomy at the segment adjacent to fusion [2–4]. Most of such potential risk factors are not modifiable and failed to fully explain the occurrence of ASD. However, biomechanical consequences of spinal fusion, even if potentially modifiable, are currently not sufficiently considered as powerful contributors to ASD. Some cadaveric studies have shown an increase in the forces and range of motion at the adjacent intervertebral joint [5, 6]. Other biomechanical consequences include the amount of stress on the adjacent disc [7], sagittal balance [8–10], pelvic parameters [11], and the amount of postoperative lumbar lordosis [12]. These biomechanical consequences of spinal fusion are identified indeed, yet not used on a patient-specific level to prove their potential contribution to ASD.

We aimed to investigate whether biomechanical changes in the lower lumbosacral region, which account for 66% of the lumbar lordosis on average [13], affect the development of ASD using a patient-specific biomechanical modeling approach. We chose to compare patients who underwent revision surgery for ASD after L4/5 single-level spinal fusion to a control group of patients without ASD after L4/5 single-level spinal fusion (CTRL group). We hypothesized that the effect of fusion surgery on musculoskeletal loads differs between CTRL and ASD patients. If so, this would imply that the risk of ASD could be determined and modified preoperatively by patient-specific musculoskeletal analyses of biomechanical consequences of spinal fusion on muscle activity and joint loads.

## Materials and methods

### Patients

The present study was a retrospective, single center, case-control study. Included were patients that underwent a primary standard open single-level spinal fusion of the L4/5 segment. All patients were initially operated on or received revision surgery at a single University Spine Center. Patients who developed degeneration and underwent revision surgery at the proximal adjacent segment L3/4 (ASD group) were compared with a cohort of control patients who did not develop postoperative degenerative changes at a minimum of 4 years follow-up (CTRL group). Excluded were patients with insufficient quality of X-ray, meaning not showing both femoral heads, the sacral tip and at least the T12 vertebra. All of these landmarks were needed for the simulation. Furthermore, spondylolisthesis of grade 2 or higher was an exclusion criteria imposed by limitations of biomechanical simulations. Additional exclusions for the control group was an insufficient outcome at follow up, which was defined by local lumbar or radiating pain. The preoperative degeneration of the adjacent segment was measured on MRIs with the Pfirrmann [14] and Weishaupt [15] classification.

### Modeling and simulation

To determine the muscular activity and joint loads in the ASD and CTRL groups, an OpenSim (v.4.0, simtk.org [16]) musculoskeletal model was created for each included patient in the pre- and postoperative conditions. Patient-specific modeling was based on an established internal modeling workflow that has been described previously in detail [17]. Modeling included modification of a generic template model based on annotated landmarks on a lateral x-ray. The generic model represents a full body model with an articulated lumbar spine and nonlinear lumbar intervertebral stiffness properties, featuring 216 muscle fascicles for the upper body and spinal articulation. A detailed description of the template model has been published previously [18].

**Fig. 1:**
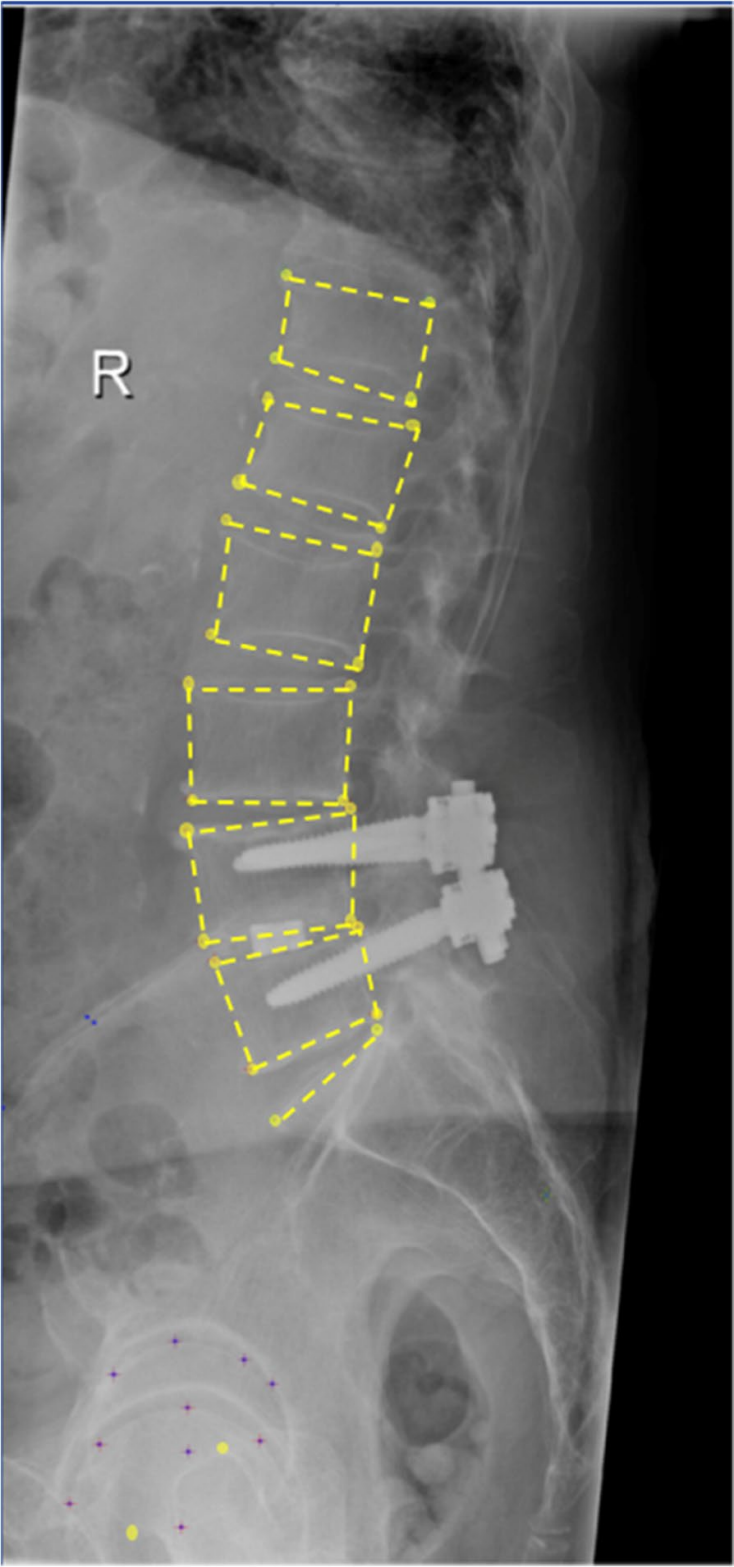
Annotation of the femoral heads, sacral endplate, and vertebral bodies on a lateral x-ray of a patient who had undergone L4/5 spinal fusion

The subsequent modeling process [11] was based on x-rays showing the following landmarks: The base- and endplate edges of the vertebrae from L5 to T12, the endplate of the sacrum and sacrum tip, and both femoral heads to obtain the midpoint of the bicoxofemoral axis (Fig. 1).

Annotations were made by two trained orthopedic surgeons on the original x-ray using a custom-built annotation tool in MATLAB (R2016b, The MathWorks, MA, USA) compiled into a standalone application. A previous evaluation of inter-rater reliability showed that the determination of vertebral body centers has an average agreement within 4.6% of vertebral body height (normalized bias), and the slope of a segment has an average agreement of within 0.4°. As the pre- and postoperative images were annotated by the same person, the intra-rater agreement was also quantified, giving average values of 3.1% for the vertebral body height and <  0.1° for the segmental slope.

**Fig. 2:**
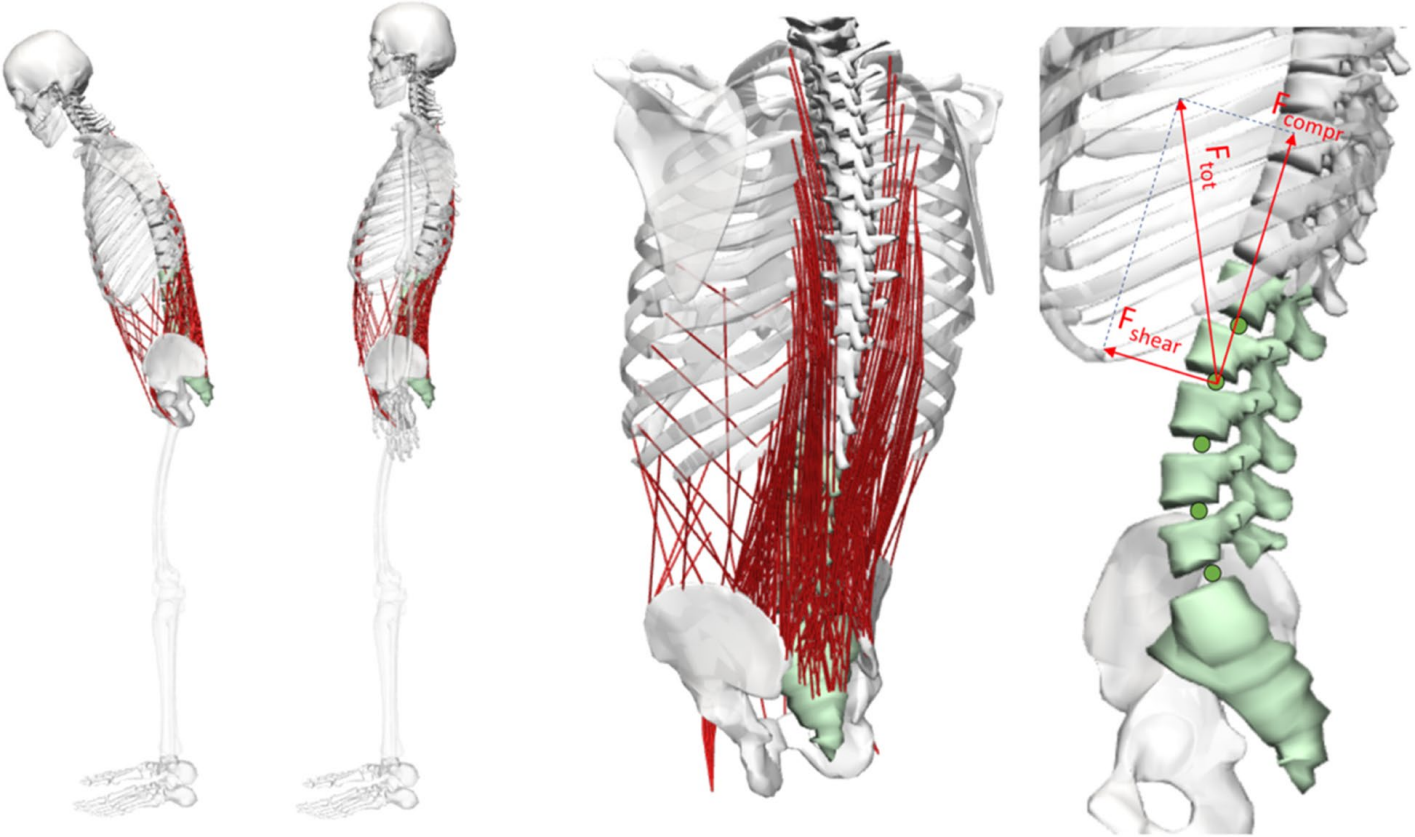
An example of our patient specific musculoskeletal model with the two postures upright and 30° foreward flexion (left). The whole trunk and the spinopelvic system with a detailed representation of the simulated musculoskeletal situation (middle). Calculated spinal forces compression and shear (right)

Each patient was simulated in the *pre-* and *postoperative* conditions in two static postures: upright standing and 30° forward flexion. The simulation itself represented a standard OpenSim workflow consisting of *Static Optimization* and *Joint Reaction Analysis*. The *Static Optimization* resolved the enforced posture into net joint moments and further into required muscle activation. The objective function for muscle activation was minimization of the overall sum of the squared muscle activation, and was comparable with minimizing the energy expenditure. The *Joint Reaction Analysis* computed the loads acting in the intervertebral joints. The computed joint loads were output as components of shear and compression. The simplification of models built on sagittal symmetry implies negligible lateral shear forces; consequently, only posterior-anterior shear and axial compression forces were assessed in the present study. (Fig. 2).

### Statistics

Postoperative muscle activity was normalized to preoperative muscle activity by 100%. A non-parametric paired test (one-sample Wilcoxon) was performed to test for intragroup differences between pre- and postoperative muscle activation. A non-parametric unpaired test (Kolmogorov-Smirnov) was performed to test for differences between the ASD and CTRL groups. Epifusional classification crosstable was calculated with fisher’s exact test. Data is presented as mean ± standard deviation. The level of significance was set to *p* <  0.05.

## Results

### Patients

The ASD group contained 20 patients, from 2004 to 2017. The mean time between the first surgery and the revision was 46 months (44-181). The CTRL group contained 18 patients, from 2004 to 2015.

There was no difference in age between the groups, 65.7 ± 8.0 years in the ASD group and 60.6 ± 13.2 years in the CTRL group (*p* = 0.154). Neither has a difference been seen in the body mass index between the two groups (Table 1). Most importantly, no difference in epifusional degeneration was seen on preoperative MRIs (Pfirrmann *p* = 0.717, Weishaupt *P* = 0.212) which were taken 1.7 months (±2.0) before the operation. Thirtyseven (97%) MRIs were analyzed, one (3%) was not available for review.

**Table 1:** Patients demographics: Data are presented as mean ± standard deviation. ASD group: patients who underwent revision surgery for adjacent segment disease after spinal fusion; CTRL group: patients without adjacent segment disease after spinal fusion

Variable	ASD	CTRL	*P* value
No. of patients	20	18	
Age (years)	65.7 ± 8.0	60.6 ± 13.2	0.154
Female sex (%)	65	50	0.363
Body mass index (kg/m^2^)	26.3 ± 3.8	26.6 ± 5.0	0.877

### Conventional measurement of spinopelvic parameters

The measured standard spinopelvic parameters (pelvic incidence, lumbar lordosis, sacral slope, pelvic tilt, pelvic incidence – lumbar lordosis mismatch) did not significantly differ between the two groups. Deviations from the calculated ideal pelvic tilt as described by Vialle et al. [19] were not significantly different between the ASD and CTRL group (Table 2).

**Table 2:** Pre- and postoperative spinopelvic parameters: Data are presented as mean ± standard deviation

	Preoperative			Postoperative		
Variable	ASD	CTRL	*P* value	ASD	CTRL	*P* value
Pelvis incidence (°)	51.7 ± 7.6	51.6 ± 11.0	0.986	54.1 ± 8.4	51.7 ± 12.1	0.499
Pelvic tilt (°)	17.0 ± 5.4	19.7 ± 7.9	0.222	19.6 ± 5.9	20.9 ± 8.2	0.605
Sacral slope (°)	34.1 ± 6.4	31.2 ± 7.4	0.198	33.7 ± 7.0	30.9 ± 6.25	0.164
Lumbar lordosis (°)	52.0 ± 9.5	47.1 ± 11.1	0.151	50.2 ± 10.3	45.2 ± 8.5	0.109
ΔPI-LL	−0.3 ± 8.9	4.6 ± 9.8	0.120	3.9 ± 9.1	6.5 ± 9.6	0.382
PTi	12.1 ± 2.8	12.1 ± 3.9	0.986	13.9 ± 3.1	12.1 ± 4.5	0.490
ΔPTi-PT	4.9 ± 4.9	7.6 ± 5.4	0.110	6.6 ± 5.1	8.7 ± 4.8	0.200

*ASD* adjacent segment disease, *CTRL* control group, *ΔPI-LL* pelvic incidence–lumbar lordosis mismatch, *PTi* ideal calculated pelvic tilt, *ΔPTi-PT* ideal pelvic tilt – pelvic tilt mismatch

### Muscular activity

**Fig. 3:**
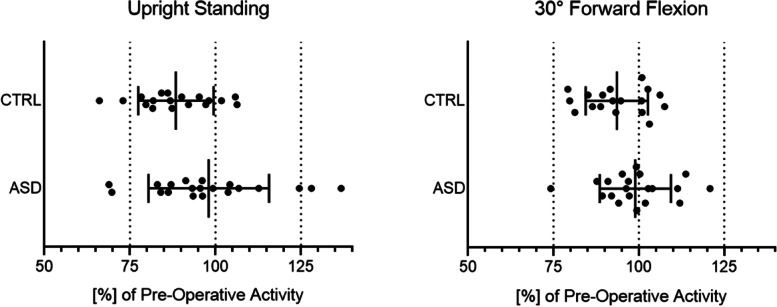
Overall postoperative muscle activity compared with preoperative muscle activity. The postoperative activation of each patient was normalized with their preoperative activation (100% - see Table 3 for values)

The CTRL group showed a significant reduction in total muscle activity from *preoperatively* to *postoperatively* in both postures; the average postoperative muscle activity was 88.4% of the preoperative activity in the upright standing posture (*p* <  0.0001) and 93.6% of the preoperative activity in 30° forward flexion (*p* = 0.016). In contrast, the total muscle activity in the ASD group did not significantly change from pre- to postoperatively in the upright standing posture (98.0%) or the forward flexed posture (99%) (Fig. 3, Table 3).

**Table 3:** Muscle activation in the upright standing (Upright) and 30° upper body forward flexed (Flexed) postures

			Upright		Flexed	
			Preop	Postop	Preop	Postop
CTRL group	Absolute activation	Mean	**5.30**	4.66*	**8.90**	**8.26**
		Range	[3.6–8.1]	[3.1–8.5]	[6.8–12.2]	[6.5–12.1]
		STD	1.32	1.28	1.56	1.24
		P	–	**< 0.001**	–	**0.020**
	Activation normalized to preop	Mean	100%	88.4%*	100%	**93.6%**
		Range	–	[66–106]	–	[79–108]
		STD	–	11%	–	9.1%
		P	–	**< 0.001**	–	**0.016**
ASD group	Absolute activation	Mean	**5.39**	**5.19**	8.53	**8.37**
		Range	[3.7–8.4]	[3.7–9.4]	[6.3–12.8]	[6.7–12.2]
		STD	1.26	1.18	1.60	1.36
		P		**0.290**	–	**0.528**
	Activation normalized to preop	Mean	100%	**98.0%**	100%	**99.0%**
		Range	–	[69–137]	–	[74–121]
		STD	–	17.6%	–	10.4%
		P	–	**0.368**	–	**0.522**

There was no significant change from pre- to postoperatively in the muscle activity of any of the individual major muscle groups. Furthermore, their activity did not significantly differ between the CTRL and ASD groups. When the muscle groups were further distinguished by anatomical locations, the activity of the multifidi muscles that attach at the fused vertebra (L4) was significantly greater in the ASD group than the CTRL group.

### Joint loads

The average joint shear loads in both the CTRL and the ASD groups differed moderately between the pre- and postoperative conditions. The forces significantly differed between the pre- and postoperative conditions in both groups at T12/L1 in the upright posture (*p* <  0.05) and at L3/4 in the flexed posture (*p* <  0.0001), as well as in the CTRL group at L2/3 in the flexed posture (*p* <  0.01) (Table 4).

**Table 4:** Shear forces in the pre- and postoperative conditions for the upright standing posture (left) and forward flexed posture (right) (Fig. 3). *P* values for non-parametric paired test between pre- and postoperative values (Wilcoxon matched-pairs signed rank). Δ(abs) is the group average of the difference in shear force magnitude between the pre- and postoperative conditions (Fig. 4)

SHEAR		Upright standing posture				30° forward flexion posture			
		preop [N]	postop [N]	p	Δ(abs)	preop [N]	postop [N]	p	Δ(abs)
CTRL group	T12/L1	− 145	− 116	0.021	- 29.0	−63	−49	0.284	- 7.7
	L1/2	− 89	−76	0.196	−12.5	−29	−26	0.966	- 0.1
	L2/3	−34	−32	0.551	- 2.6	48	32	0.004	- 8.1
	L3/4	30	34	0.417	- 4.6	76	37	< 0.0001	- 38.4
	L5/S1	309	302	0.610	- 6.9	401	403	0.966	- 8.7
ASD group	T12/L1	− 180	− 155	0.044	- 24.4	−93	−78	0.430	- 8.0
	L1/2	−101	−90	0.246	- 9.5	−37	−31	0.870	- 11.7
	L2/3	−41	−36	0.498	- 4.9	43	32	0.064	- 11.6
	L3/4	39	44	0.246	+ 3.9	90	49	< 0.0001	- 39.5
	L5/S1	356	348	0.189	- 8.6	445	436	0.409	+ 2.0

The average shear loads in the ASD group tended to be slightly larger than those in the CTRL group. However, significant differences were only detected between the groups in the forces at L5/S1 during upright standing in both the pre- and postoperative conditions (*p* = 0.024 and *p* = 0.026, respectively).

**Fig. 4:**
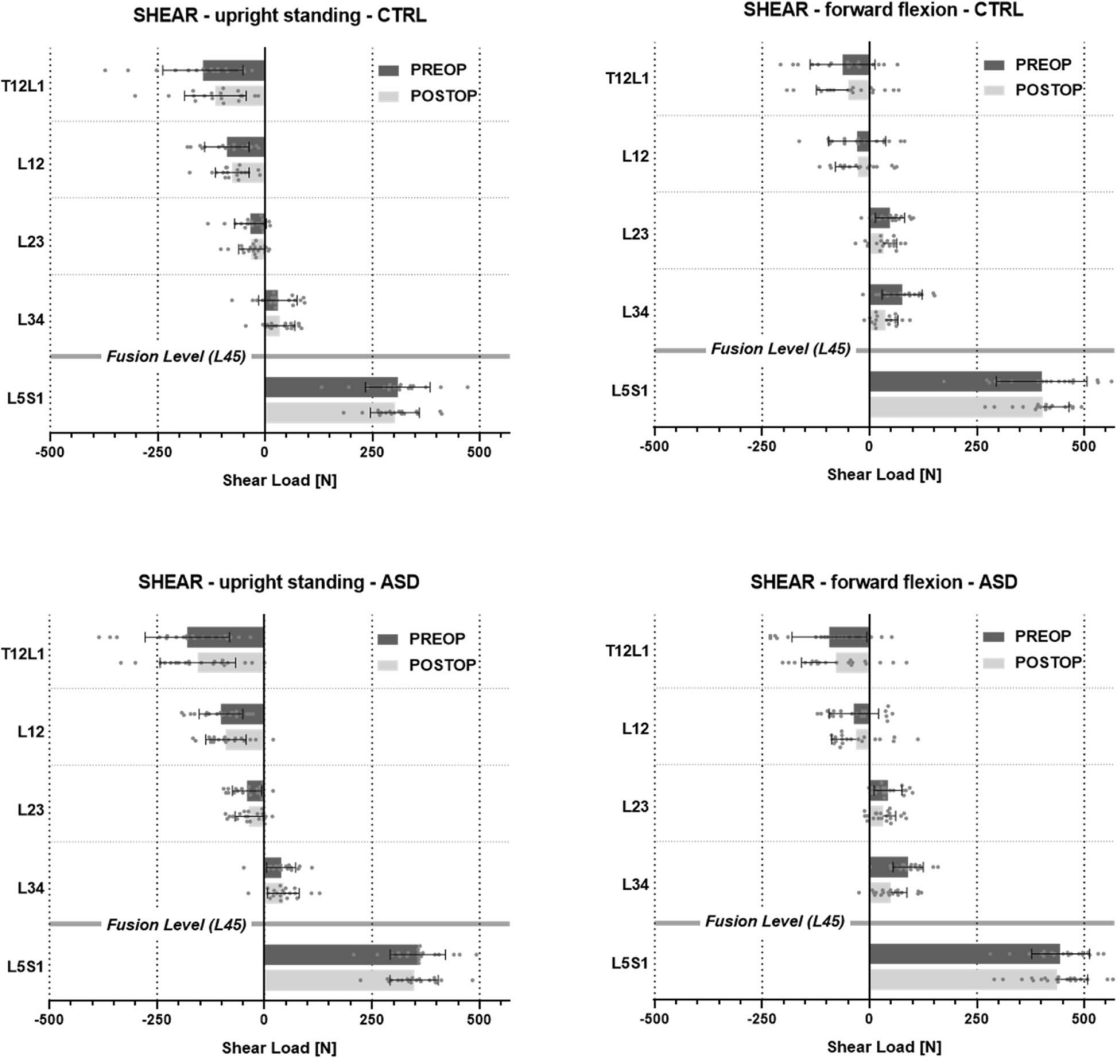
Shear forces in the pre- and postoperative conditions in the upright standing posture (left) and forward flexed posture (right) for the control (CTRL) and adjacent segment disease (ASD) groups (top and bottom, respectively)

When the changes in shear load magnitudes from the pre- to postoperative condition were considered on a patient-by-patient basis, the effect of fusion on shear loads was similar in both the CTRL and ASD groups. (Fig. 4).

**Fig. 5:**
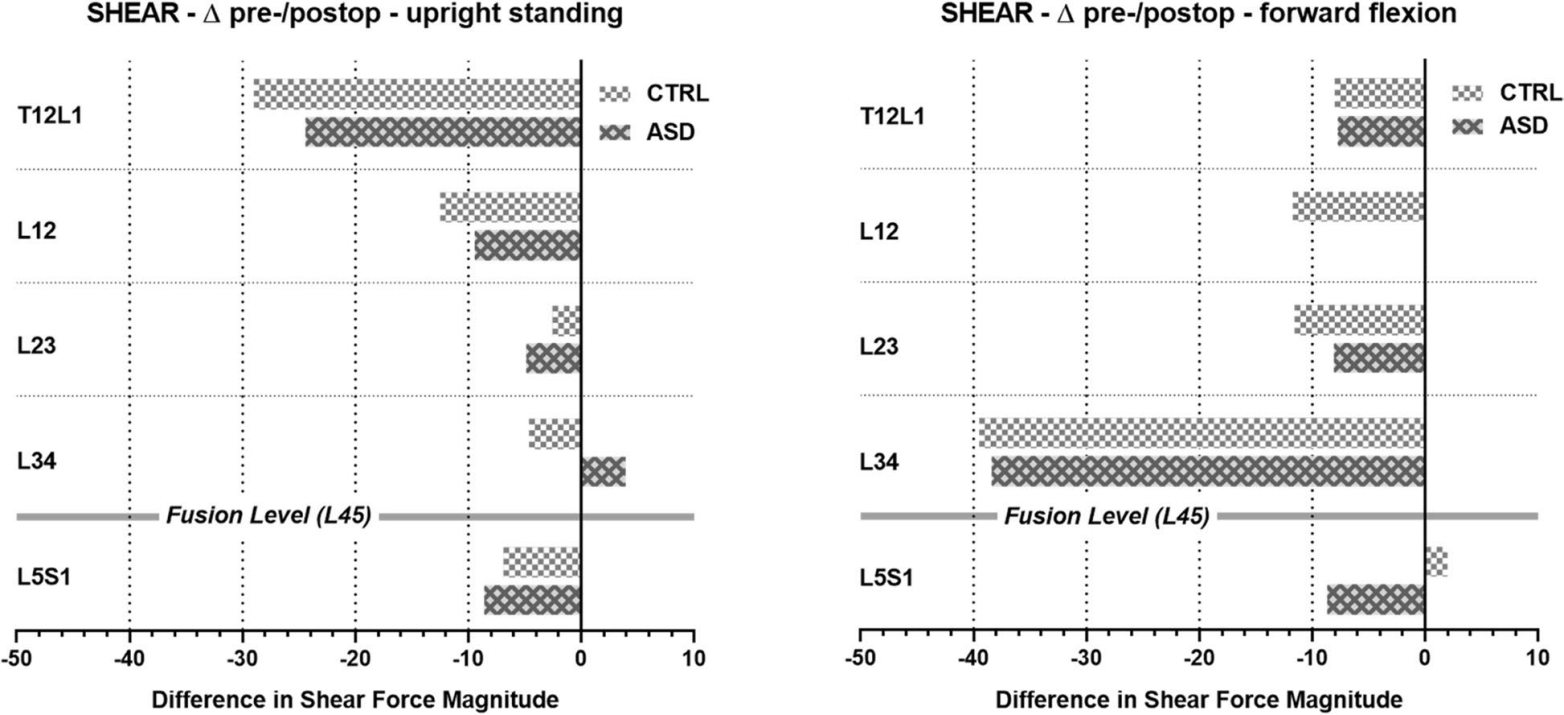
Average difference in shear force magnitude between the pre- and postoperative conditions. The pairs of bars represent the control (CTRL; light grey, top) and adjacent segment disease (ASD; dark grey, bottom) groups

However, opposing trends were observed at the epifusional joint L3/4, which experienced a postoperative increase in shear load magnitude in the ASD group during upright standing (+ 3.9 N) and a postoperative decrease in the CTRL group (− 4.6 N) (Fig. 5). Similarly, the force at L5/S1 slightly increased from pre- to postoperatively in the ASD group during flexion (+ 2.0 N), but decreased from pre- to postoperatively in the CTRL group (− 8.7 N). However, although the average reduction in shear force magnitude was larger in the CTRL group than in the ASD group, these pre- to postoperative differences were not significant in either group.

## Discussion

Among many potential factors influencing the development of ASD, biomechanical factors are modifiable by the surgeon, however yet not fully understood. Patient-specific musculoskeletal analyses of biomechanical consequences of spinal fusion could have predictive merits towards development of ASD and modify clinical decision making and surgical planning. Comparing the biomechanical effects of fusion on a patient-specific level allowed us to reveal important differences of patients with ASD versus without.

This concept is in concordance with several other studies that have shown that spinal fusion may result in an increase in motion and forces in the epifusional segment [5, 6, 20] based on cadaveric or finite element investigations. Abode-Iyamah et al. found significant increasing disc pressure in the adjacent segment in a cadaveric study of nine specimens after lumbar spinal fusion [21]. Forces reported in such studies often rely on intradiscal pressure measurements, which are known to correlate with compressive loads in the discs. However, *shear* is an important loading mode in the context of onset and progression of disc degeneration [22, 23]. The loading of intervertebral segments in general is predominantly a result of body weight and active force contribution due to muscular activity. The separation of overall loads into shear and compressive load components are mainly a result of the orientation of the disc, and thus dependent on segmental kinematics during body movement. While cadaveric studies and most finite element studies do not assess the active load contribution of muscles, musculoskeletal modeling and simulation is capable of integrating muscle forces into analyses [17, 18, 24]. Therefore, the present study used the approach of patient-specific *musculoskeletal analysis* to address the hypothesis that muscular activity and intervertebral loading in patients with ASD differ from those in asymptomatic controls.

In the present study, two very homogenous groups were compared, initially having the same level fusion, the same demographic factors and did not show any difference in pre- and postoperative spinopelvic parameters nor in the Roussouly classification. The here demonstrated results confirmed the hypothesis that the asymptomatic CTRL group had less total activity of the core- and paraspinal muscles after L4/5 spinal fusion compared with the activity prior to fusion. In contrast, the ASD group demonstrated unchanged muscular activity. As the loading of intervertebral joints largely depends on muscular activity, these results also suggest the presence of larger and potentially adverse loading in patients who developed ASD. The increased postoperative muscle activity in the ASD group as compared to the CTRL group during upright standing may also be explained by a less balanced upper body posture in the ASD group, which requires higher muscle forces to maintain equilibration. Therefore, as expected, the ASD group also had greater compressive forces in the upright standing posture as compared to the CTRL group. The finding of this simulation may be used in preoperative planning to find the best possible sagittal alignment for every patient in order to have the least amount of muscle activity and compression forces after surgery. Hence, the presented method offers a possibility to surgeons to preoperatively simulate and calculate the optimal fusion parameters for each patient, in order to achieve lower loadings and reducing the risk of ASD. Excitingly, a difference between the two groups was seen in our biomechanical analysis, which could not be seen with the established conventional spinopelvic parameters. Thus, a more accurate and precise preoperative planning might be made with the presented simulation as compared to the conventional preoperative planning currently used for the majority of surgical planning.

As with any patient-specific simulation study, the modeling process was governed by simplifications and assumptions. General limitations are presented and discussed in detail in previous publications [17, 24]. The following specific limitations were identified for the present cohort study: First, the muscle properties and body masses could not be fully individualized. Although this means that the models were less personalized, this method was justified because it enabled the comparison of results between patients and groups without normalization. Also, the spinopelvic anatomy, which is considered the major risk factor for ASD, was specifically represented in all models. It was individualized based on lateral X-rays, with the inherent blurring of the beam path. The projection angle of the spinopelvic system was adjusted and the beam magnification balanced by the bicoxofemoral axis. After adjustment, the effects of X-ray imaging on spinal alignment were considered minimal. On the contrary, conventional radiographs had a remarkable advantage over other imaging modalities such as CTs: the standing posture of the patients. This is more representative to loading scenarios in daily living, as well as more accurate for the assessment of spinopelvic alignment, thus better justifying comparison to the existing literature. Second, the assumed segmental kinematics for obtaining the flexed postures were generic, and therefore the simulated slope angles of intervertebral discs in the flexed posture may have deviated from reality. Although this is not particularly relevant for muscle activity and total joint loads, it affects the breakdown of loads into shear and compression. Despite recent efforts to successfully quantify the motion of vertebrae in vivo [25], there is currently no method allowing continuous and systematic assessment of spinal kinematics. Consequently, it is necessary to use a generally assumed spinal kinematic rhythm when simulating postures for which no radiological data exist. Third, given the variability of anatomy and conditions of spinal fusion patients in combination with the multifactorial pathogenesis of ASD, the patient population included in the present retrospective study was fairly limited. Future investigations including other single- and multi-level fusions as well as prospective studies are likely to provide even clearer separations between groups. And last, the degeneration of the adjacent segments was not taken into consideration with this simulation, yet we know about the change of forces with the increasing degeneration of a segment [26]. But since the patients of the two groups did not have a difference between the preoperative degeneration of the adjacent segment this factor might in our case be neglected.

The differences of the forces between the groups were in many measures very small. One can imagine that these parameters gain significance with increased body weight, especially around the waist and trunk as well as with more physical work. These may also be risk factors and influence the occurrence of an ASD but in our case had no significant difference between the groups. The total muscle activation is also a sign of a worse osseous balance, which is reinforced with additional body weight and with physically demanding work.

The study limitations explain, to some extent, the clearer separation between the present ASD and CTRL groups for muscle loads than for joint loads. The muscle activity is mainly a direct result of the posture and anatomy. In contrast, the shear and compression components depend on the muscle activity, as well as on the anatomy and kinematics. The high sensitivity of load components to disc orientations, which are modeled with limited accuracy, may thus reduce the intergroup differences in shear forces.

## Conclusion

The current study showed that in patients with ASD at L3/4, the L4/5 lumbar spinal fusion did not increase the overall muscle activity. In contrast, patients who had undergone spinal fusion at L4/5 and had not developed ASD after a minimum 4 years of follow-up showed a significant reduction in overall muscle activity compared with preoperatively. The present results therefore provide the basis for future studies aiming for a prognostic biomechanical evaluation of the risk of ASD in patients undergoing spinal fusion surgery. Geometrical planning, being currently the standard of surgical planning, neglects the emerging possibilities of considering biomechanical and kinematic information in a patient-specific way. A change of paradigm in evaluation of risk of ASD on a patient specific level using musculoskeletal biomechanical planning is described here and should introduce further research and development towards the next level of surgical planning.

## Data Availability

The datasets used and/or analyzed during the current study are available from the corresponding author on reasonable request.
